# Wind Direction and Its Linkage with *Vibrio cholerae* Dissemination

**DOI:** 10.1289/ehp.9391

**Published:** 2006-10-18

**Authors:** Shlomit Paz, Meir Broza

**Affiliations:** 1 Department of Geography and Environmental Studies, University of Haifa, Haifa, Israel; 2 Department of Biology, Faculty of Science and Science Education, University of Haifa, Oranim, Tivon, Israel

**Keywords:** Africa, Chironomidae, India, *Vibrio cholerae*, wind

## Abstract

**Background:**

The relevance of climatic events as causative factors for cholera epidemics is well known. However, examinations of the involvement of climatic factors in intracontinental disease distribution are still absent.

**Objectives:**

The spreading of cholera epidemics may be related to the dominant wind direction over land.

**Methods:**

We examined the geographic diffusion of three cholera outbreaks through their linkage with the wind direction: *a*) the progress of *Vibrio cholerae* O1 biotype El Tor in Africa during 1970–1971 and *b*) again in 2005–2006; and *c*) the rapid spread of *Vibrio cholerae* O139 over India during 1992–1993. We also discuss the possible influence of the wind direction on windborn dissemination by flying insects, which may serve as vectors.

**Results:**

Analysis of air pressure data at sea level and at several altitudes over Africa, India, and Bangladesh show a correspondence between the dominant wind direction and the intracontinental spread of cholera.

**Conclusions:**

We explored the hypothesis that winds have assisted the progress of cholera Vibrios throughout continents. The current analysis supports the hypothesis that aeroplankton (the tiny life forms that float in the air and that may be caught and carried upward by the wind, landing far from their origin) carry the cholera bacteria from one body of water to an adjacent one. This finding may improve our understanding of how climatic factors are involved in the rapid distribution of new strains throughout a vast continental area. Awareness of the aerial transfer of *Vibrio cholerae* may assist health authorities by improving the prediction of the disease’s geographic dissemination.

Cholera continues to be an important cause of morbidity and mortality in many areas of the world, and there is currently a high frequency of new outbreaks in Africa. The fatal effects of the disease are mainly due to the cholera toxin produced by specific strains of *Vibrio cholerae*.

In 1817, cholera spread out of its endemic distribution on the Indian subcontinent and reached many countries around the world, appearing as a global event, or pandemic. In 1961, *Vibrio cholerae* O1 biotype El Tor spread out of Indonesia toward India, replacing the O1 “classical biotype,” and creating the “seventh pandemic.” During summer 1970, it continued to spread westward through the Middle East into Africa. The seventh pandemic began in Africa in August 1970 on two fronts, simultaneously striking the north and the west, with devastating consequences. Cholera quickly spread throughout the continent. Inadequate resources and limited access to health care led to high death rates (Swerdlow and Isaäcson 1994).

In October 1992 a new serogroup, now known as O139, caused a severe outbreak of cholera in the city of Madras, in southeast India. Within 10 months, the O139 serogroup had disseminated all over the Indian subcontinent and soon thereafter spread to 11 neighboring countries in Asia and beyond, temporarily replacing the O1 serogroup as the most common (Ramamurthy et al. 1993). What could enable such rapid distribution of the *Vibrio cholerae* O139 throughout the subcontinent? The answer is still a mystery.

While maps and reports describing the El Tor pandemic of 1970 showed its spread on a continental and global scale [e.g., Todar 2005; World Health Organization (WHO) 2000], researchers in India were able to track the replacement of the O1 by O139 serogroup in town after town, over a period of 10 months (Nair et al. 1994). However, both the spread of El Tor in 1970–1971 and O139 in 1992–1993 show a tendency for wave after wave of gradual westward movement. Although person-to-person contact plays an important role in the rapid spread of cholera, especially in crowded religious festivals and on all types of modern public transportation, these pandemics included cases that cannot be explained by any human activity or mechanical transportation. In some other cases, epidemics appeared simultaneously in remote unconnected locations.

Chironomids, the nonbiting midges (Diptera; Chironomidae), are one of the most abundant macroinvertebrate groups that exist in most freshwater aquatic habitats (Armitage et al. 1995), especially in estuarine (Benke 1998) and organic-rich water bodies typical to *Vibrio cholerae.*

The eggs, larvae, and pupae are aquatic; adults emerge into the air for mating and dispersion. Chironomid adults are highly mobile, present in very large numbers, and found frequently in the air at various elevations, as was noted by Glick (1939) and more recently by Reynolds et al. (1999) (see also Bowden and Johnson 1976; Sparks et al. 1986).

Broza and Halpern (2001) reported that the huge number of chironomid egg masses attached to hard substrates on the water surface serve as a reservoir for the free-living *Vibrio* bacteria. Also, Broza et al. (2005) observed that both male and female chironomids, while emerging from the water, may carry cholera bacteria into the mating swarms. In experimental simulation, they demonstrated that the cholera-bearing adult midges are carried by the wind, and transmit the bacteria from one body of water to another. Broza et al. (2005) suggested that flying adult midges can act as a vector for cholera in continental and even intercontinental spread. However, this hypothesis has not yet been tested in a true cholera epidemic or pandemic.

Much information is available regarding the relevance of climatic events as causative factors for cholera epidemics. Several studies (e.g., Huq et al. 2005; Lipp et al. 2002) focused on the effect of the main climatic factors on the initial eruption of cholera, such as precipitation, air and water-surface temperature, and relative humidity. These studies did not, however, address the climatic connection to disease dissemination over vast continental areas.

Because winds are the driving force behind the long-distance flight of insects, we suggest exploring the linkage between wind direction on a local and global scale and the sequence of epidemic dissemination. In the present study, we examine three cases of cholera outbreaks that occurred in Africa and on the Indian subcontinent within the last 35 years, focusing on linkages between dominant wind direction and the disease dissemination.

The African monsoonal winds are ultimately caused by the differential heating of land and ocean, which inevitably leads to a pressure gradient. Atmospheric circulation transports very different types of air masses, whose presence results in the occurrence of diametrically different wet and dry seasons. The circulation over West Africa is typically monsoonal with seasonal change in wind direction. The humid Guinea monsoon blowing from the southwest moves northward behind the convergence zone, and by April it reaches the borders of Mali and Niger. In eastern Africa, the seasonal exchange of air masses flow from opposite directions, from parts of continental Africa in the northern and southern hemispheres. In April, Indian Ocean trade winds blow from the sea to the coastline and the mountain ranges. In East and West Africa and in the interior areas of the equatorial zone, the first of two annual precipitation peaks occurs in April and May, whereas the second peak occurs in October and November with lower amounts of precipitation [Intergovernmental Panel on Climate Change (IPCC) 1996; Martyn 1992].

Climatic patterns in southern Asia depend primarily on the geographic position in tropical and equatorial latitudes. The Indian climate is dominated by the monsoon, which shows spatial, interannual, and intraseasonal variability. The monsoon is characterized by a reversal in the winds over the Indian subcontinent between summer and winter. In winter the winds blow off the continent to the southwest, and in summer (starting in May) they blow from the southeast. The wind shift is associated with heavy rains over India in the summer. After the withdrawal of the summer monsoon, the northeast monsoon sets in by November and continues through December. Due to very active weather systems in the Bay of Bengal, many cyclones occur during this season, mainly affecting the eastern coast of India [Martyn 1992; National Oceanic and Atmospheric Administration (NOAA) and Climate Diagnostic Center 2004].

We hypothesize that a potential aerial transfer of *Vibrio cholerae* over vast continental areas is related to a correlation between spatial incidence patterns and wind direction. In the present study we evaluated three cholera outbreaks within the seventh and the eighth pandemics: the disease dissemination over parts of Africa throughout 1970–1971; the disease dissemination in the same areas in 2005–2006; and the *Vibrio cholerae* O139 rapid spread over the Indian subcontinent during 1992–1993. We evaluated the dissemination of these epidemics through wind direction analysis (the dominant wind direction in a selected region computed based on monthly synoptic maps), examining the possible influence of wind direction on flying insects carrying viable cholera bacteria attached to their cuticle.

In this study, we also investigated the hypothesis of Broza et al. (2005) that aeroplankton may carry the cholera bacteria from one body of water to an adjacent one. Aeroplankton are the tiny life forms that drift through the air, are carried upward by the wind into the atmosphere, and continue to be carried by the wind for a considerable length of time, landing far from their origin. Small organisms such as flies may be part of the aeroplankton. They may be driven by strong winds in lower altitudes during their swarming behavior at a height of 5–100 m.

## Methods

### Data for Africa, 1970–1971

We obtained data for cholera dissemination in Africa for 1970–1971 from Swerdlow and Isaacson (1994) and for October 2004 to March 2006 from the World Health Organization (WHO 2005, 2006). To create wind direction maps for Africa [using National Centers for Environmental Prediction–National Center for Atmospheric Research (NCEP-NCAR) Reanalysis Project database and software (NOAA NCEP-NCAR 2006], we used mean monthly sea level pressure [SLP; in millibars (mb)] and mean monthly geopotential values for three altitude levels: 1,000 mb (150 to ~ 200 m), 925 mb (700 to ~ 800 m), and 850 mb (~ 1,500 m) covering the whole African continent (NOAA NCEP-NCAR 2005). These values were measured for each month from July 1970, one month before the West African cholera epidemic appeared in Guinea, until December 1971, the last month of the epidemic in Africa (NOAA NCEP-NCAR 2005). The altitude levels are in accordance with those examined for aeroplankton by Glick (1939).

### Data for Africa, 2005–2006

We also mapped the same SLP, 1,000 mb, 925 mb, and 850 mb levels (NOAA NCEP-NCAR 2005) for Africa for each month from October 2004, when *Vibrio cholerae* O1 El Tor was confirmed in Senegal, to March 2006 (cholera has been reported in southern Sudan).

### Data for India

Our study of India was based on a summary of the temporal and spatial spread of O139 during 1992–1993 by the National Institute of Cholera and Enteric Diseases in Calcutta, India (Nair et al. 1994). We analyzed mean monthly SLP and mean monthly geopotential values for 1,000 mb, 925 mb, and 850 mb levels, covering the subcontinent of India and Bangladesh for each of the 10 months from October 1992 (when the *Vibrio cholerae* serogroup O139 caused an outbreak in Madras) to July 1993 (when the disease reached northwestern India). We produced wind-direction maps using the NCEP-NCAR Reanalysis Project database (NOAA NCEP-NCAR 2006). Anomalies from the monthly averages, based on the 1951–2000 period, were also mapped and analyzed.

### Correlations

In the present study, the dominant wind direction refers to the prevailing winds over a selected area during a chosen month using monthly average pressure maps at different altitudes. The main direction was identified based on the principles for the Northern Hemisphere—that the wind blows counterclockwise around a low-pressure area and clockwise around a high-pressure area. Although the wind behavior is characterized by a rapid change in direction and speed, the maps present the most expectable wind direction throughout the month.

To quantify the correlation between wind direction and cholera spatial dissemination pattern for *Vibrio cholerae* dissemination in Africa during 1970–1971, we used the formula for circular correlation (ρ*_circ_*) of Jammalamadaka and SenGupta (2001):


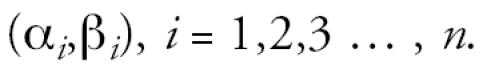


This formula denotes the sample pairs of directions (wind direction and the cholera spatial dissemination direction). The formula for the general sample correlation is


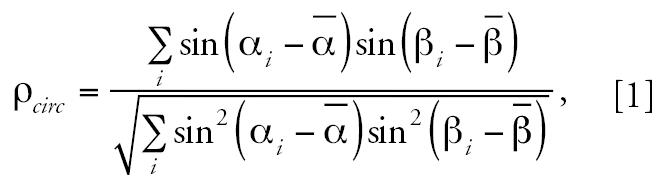


where 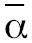 is the sample mean defined as


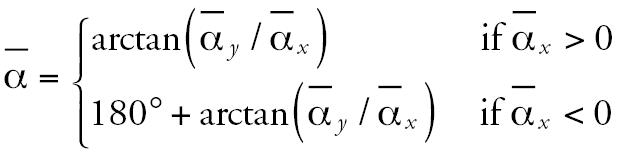


and


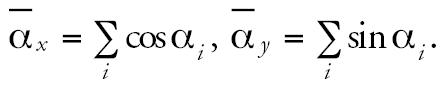



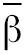
 is defined similarly. The ρ*_circ_* ranges between −1 and 1, similar to the linear correlation.

## Results

### Africa

**Figure 1:**
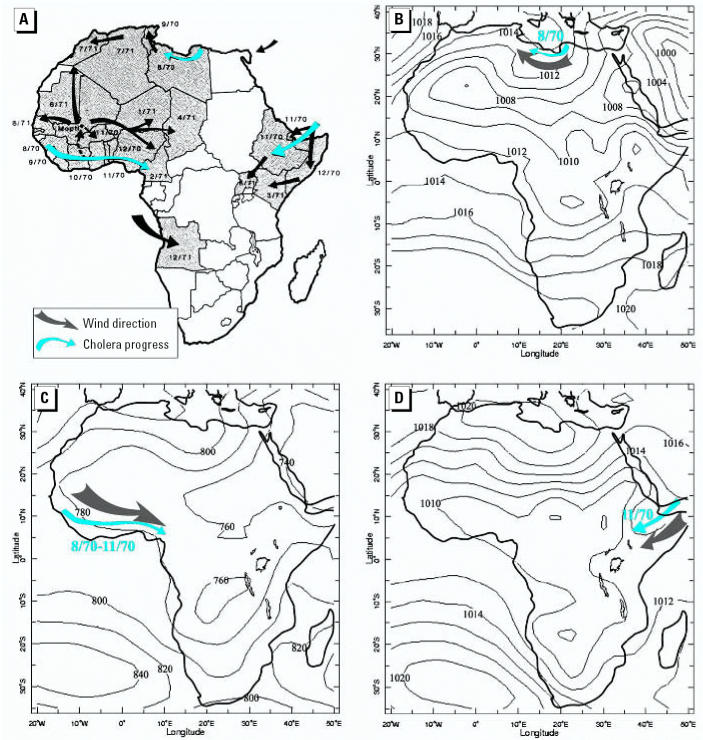
Maps showing movement of cholera in Africa during 1970–1971 (adapted from Swerdlow and Isaäcson 1994) (*A*) and the dominant wind direction in Africa in SLP (in millibars) in August 1970 (*B*), air pressure of 925 mb level in September 1970 (*C*), and SLP in November 1970 (*D*). Black arrows in (*A*) indicate other waves of cholera movement. Values in (*C*) indicate altitude in meters.

Figure 1A–D shows examples of the results of mapping monthly average pressure values in the different altitudes over Africa during summer and autumn 1970. Note the impressive overlap between the dominant wind direction and the cholera progress in North Africa (August 1970), West Africa (September 1970), and East Africa (November 1970). Although we have not attempted to explain the disease spread throughout the entire area in 1970–1971 (e.g., the spreading from Mopti, the pilgrimage site in Senegal, could be a result of human dissemination), the influence of wind direction on the cholera dispersion adds a new explanation for disease dissemination in key parts of the continent.

**Figure 2:**
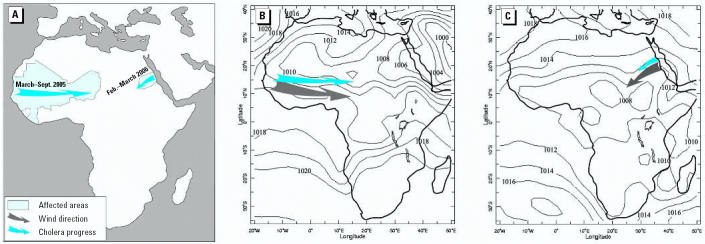
Progression of cholera over Africa in March–September 2005 and February–March 2006 [*A*; adapted from WHO (2005) and WHO (2006)] and the dominant wind direction (shown by SLP maps in millibars) over the continent in July 2005 (*B*) and February 2006 (*C*).

Moreover, we obtained the sample circular correlation using 11 paired direction measurements from the *Vibrio cholerae* progress in Africa during 1970–1971 for the stages presented in Figure 1B–D. Our result is ρ*_circ_* = 0.958 (*p* = 0.006), indicating an impressive significantly high positive correlation. Figure 2 shows the relationship between the geographic dissemination of cholera over western and northeast Africa during March–September 2005 and February–March 2006 and the dominant wind direction over the continent in the same periods. Figure 2 shows a convincing correspondence (see selected examples in Figure 2A–C).

### India

**Figure 3:**
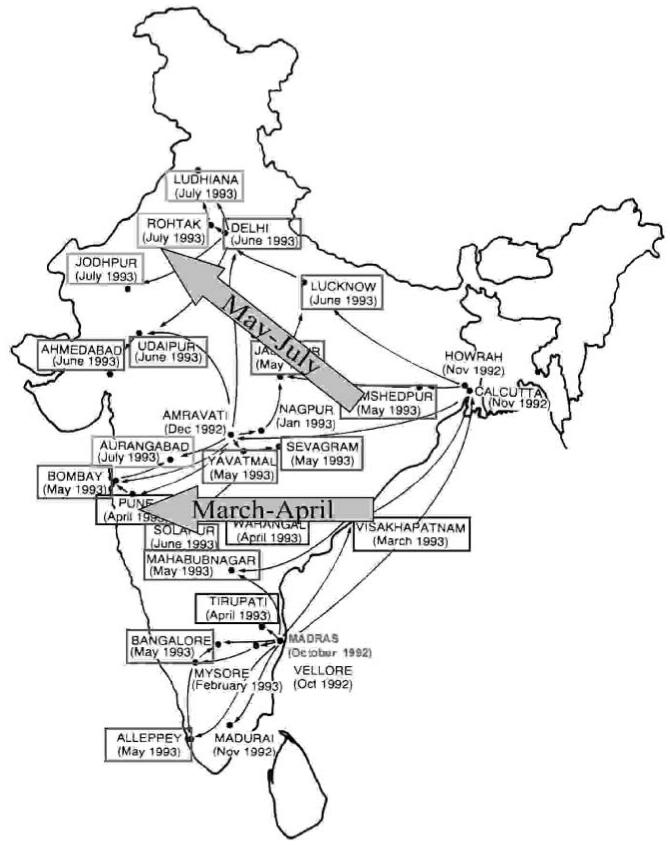
Spread of *Vibrio cholerae* O139 in India from east to west (adapted from Nair et al. 1994). An arrow shows the dissemination of cholera from east (Visakhapatnam) to west (Pune) during March–April 1993; this spread of cholera continued from May to July 1993. A second arrow indicates the spread of the epidemic upstream along the Ganga River Valley in May–July 1993.

**Figure 4:**
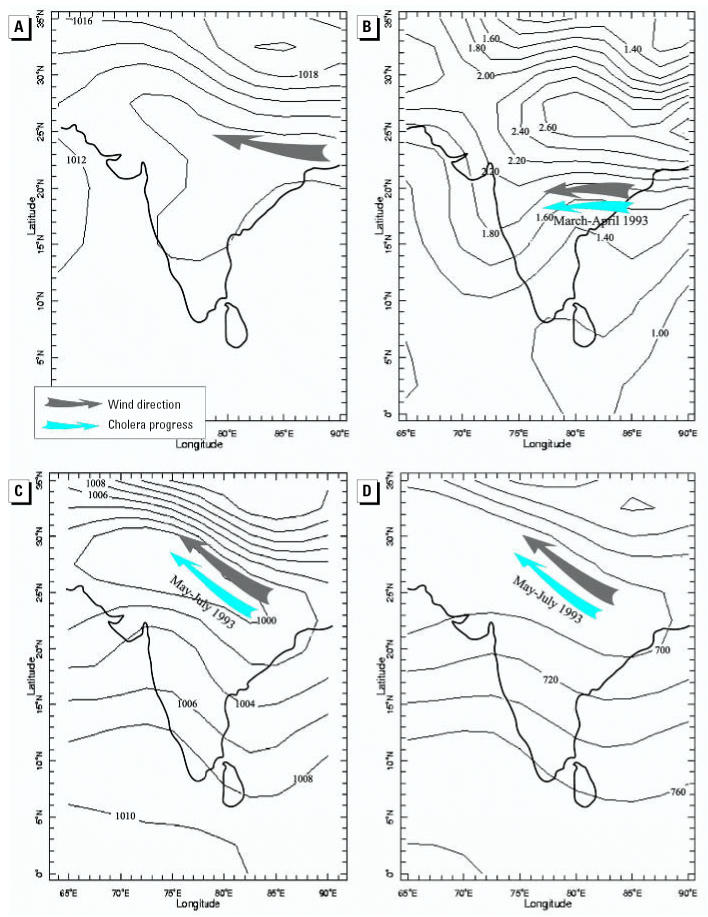
Maps showing (*A*) mean SLP (in millibars) for 1951–2000; (*B*) deviations in SLP for March 1993 calculated based on the 1951–2000 averages for March; (*C*) mean monthly SLP for June 1993; and (*D*) dominant wind direction over northern India during June 1993 at the 925 mb level. Values in (*D*) indicate altitude in meters. Note how the geographic dissemination of *Vibrio cholerae* O139 corresponds to the dominant wind direction.

During March and April 1993, the air pressure values in the eastern part of India were significantly higher (by *t*-test analysis) than the average values for 1951–2000 (Figures 3 and 4A–B), and therefore caused a strengthening of the eastern winds. Table 1 presents the significant deviation of the mean SLP values in March 1993 from the mean values of 1951–2000, as well as the *t*-test results (*p* ≤ 0.05) for selected grid points. In addition, after averaging all standardized deviations per year, we found that March 1993 was the month with the second-highest positive deviations throughout the entire 50 years, with an average *t*-score of 1.52.

These extreme easterly winds could carry the cholera bacteria from the east coast to Pune in the west. Indeed, there is an overlap between the wind direction and the cholera progress from eastern India to western India in March–April 1993 (Figures 3 and 4A–B).

To observe the diseases spreading in northern India, we compared the progress of cholera in May, June, and July (Figure 3) with the wind patterns at the SLP (an example is presented in Figure 4C). Geographic dissemination of *Vibrio cholerae* O139 completely corresponds to the dominant monthly wind direction, from the east (Jamshedepur) to the northwest.

Results of mapping monthly average geopotential values at the three different altitudes (1,000 mb, 925 mb, and 850 mb) together with their anomalies show the same trends as were detected from the SLP maps. The results show an overlap between the disease progress in the spring and the extreme easterly winds in March 1993. The results also show the dissemination of cholera toward the northwest in May–July 1993 to correspond with the southeasterly winds over northern India. Figure 4D shows a selected example of the wind directions at the upper levels.

## Discussion

In the present study we compared the progression of cholera in three epidemics in Africa and India with the prevailing winds over the relevant areas. The use of synoptic maps supports our hypothesis that the cholera-bearing adult midges are carried by the wind and transmit the bacteria from one body of water to another over large areas.

*Vibrio cholerae* bacteria are common hitchhikers attached to the surface of adult nonbiting midges (observed mainly with *Chironomus* sp., family Chironomidae). Both males and females have been reported to carry *Vibrio cholerae* strains that remain viable and culturable even after 14 days (Broza et al. 2005).

**Figure 5:**
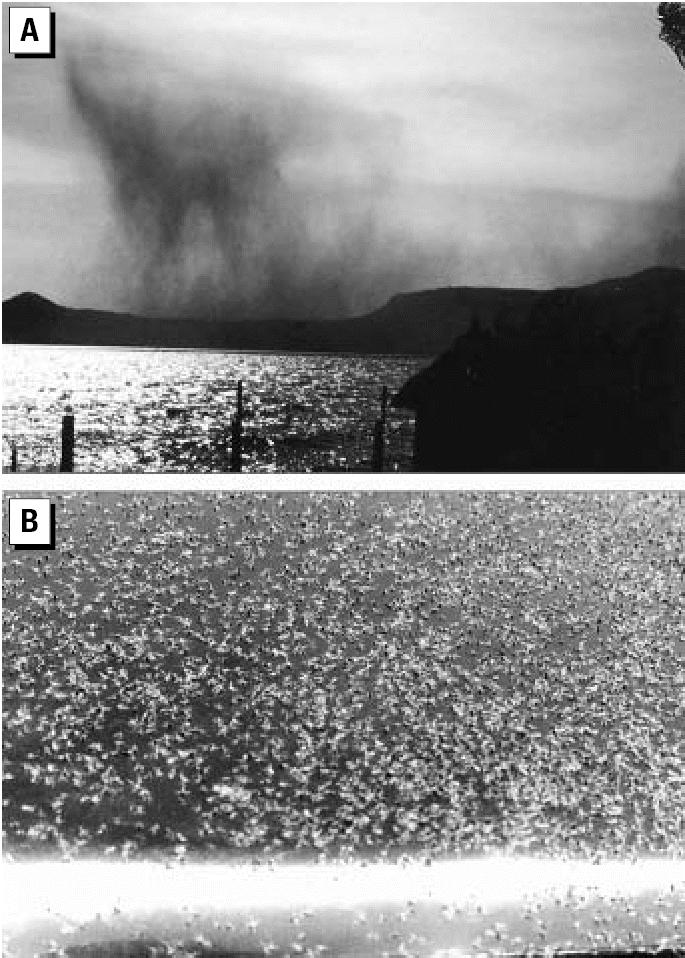
Photographs of (*A*) sunset on Lake Victoria (Kenya) showing a huge swarm of nonbiting midges moving over Rusinga Island toward the northern shore of the lake (photographed by H. Nadel), and (*B*) adult chironomids adherred to the engine cover of a car passing beside a sewage pond before sunrise (photographed by M. Broza).

At sunset on 5 June 2000 (Figure 5A), a huge swarm of nonbiting midges (Diptera; family Chaoboridae), mixed with a minor percentage of Chironomidae, were photographed near the northern shore of Lake Victoria in Kenya. Soon thereafter, adult midges were swept offshore by a strong north wind combined with heavy rains typical of a monsoon period. At M’bita Point Experimental Station, millions of adult midges moved through the air and immediately adherred to the wet surface of maize plants, trees, walls, and parked vehicles (Figure 5B). It was a “cholera time” along the lakes of the African Rift Valley, and even in M’bita Point a few people were infected with cholera. A sample of adult flies was found to be positive for *Vibrio cholerae* bacteria (Broza et al. 2005).

Atmospheric transports of pathogens, harbored in the body fluids of their insect vectors, have been recorded repeatedly in the last two decades (Ritchie and Rochester 2001; Sellars et al. 1982; Tissot-Dupont et al. 2004). However, the potential of adult nonbiting midges to carry aquatic *Vibrio cholerae* bacteria (attached to their chitinous surface, mainly on the intersegmental membranes) was only recently suggested (Broza et al. 2005).

In Africa, the seventh pandemic seriously affected populations to a greater extent than many other local and global diseases, and second or third waves of cholera have continued occurring through early 2006. The worst wave of all occurred in the heart of Africa, in Rwandan refugee camps in the town of Goma (1994, 1997, and 2001) (WHO 2000). This cholera pandemic started in 1970–1971, at the perimeter of the African continent along its northeastern, northern, and western shores. Although other important factors contribute to the spread of disease (e.g., pilgrims), we suggest that the dissemination of the seventh pandemic in Africa was related to the synoptic conditions over the continent, because the wind directly affected the distribution of the cholera-contaminated flying insects, which are part of the aeroplankton.

The event that led to the emergence of the new pathogenic serogroup of *Vibrio cholerae* (O139) in the Indian subcontinent was probably caused by two independent clones (Faruque et al. 2003; Nair et al. 1994): one on the southeastern corner of the Bay of Bengal (Chennai to Vellore) and the other in the northeastern part (Calcutta to Dhaka, Bangladesh). As noted by Colwell (1996), in many cases the outbreak started at the seashore interface, usually in an estuarine area, that in most cases was created by a river delta (e.g., Cavery Delta in southern India and the Ganges delta in the northwest).

An analysis of the spread of O139 throughout India over 10 months allows us to divide the process into a number of distinct phases.

- During a short monsoon period in October–December 1992, the O139 epidemic was established in the south (Chennai–Vellore–Madurai area) and immediately thereafter in the Calcutta–Howrah–Amravaty area (November–December).
- During an intermonsoon period, the epidemic spread throughout the center of India. From a biological viewpoint, the epidemic might be related to the fact that March–April is usually an interepidemic season. However, in relation to our present synoptic analysis, the O139 epidemic is significantly correlated to the abnormal deviation of the SLP values during spring 1993, causing a significant strengthening of the easterly winds and therefore encouraging the cholera distribution from east to west.
- During a long monsoon period in May–July 1993, cholera’s progress up the river was speeded up by the wind, all the way from Calcutta to northwest India (Jodhpur, July 1993). This last phase coincided with the usual timing and location of cholera in the Ganga Valley, which indicates a correlation between wind direction and cholera dissemination. Overall, the first O139 epidemic (sometimes called the eighth pandemic) did not follow the “seasonal” pattern of cholera occurrence in India but occurred over 10 consecutive months.

## Conclusions

In a recent study, it was suggested that aeroplanktonic adult flies may carry the cholera bacteria between bodies of water (Broza et al. 2005). The aim of the present study was to further support this claim by showing linkages between wind (the vehicle of aeroplankton) and cholera-spreading patterns. Indeed, the present data connects wind direction with cholera dissemination patterns. Moreover, there is a clear relationship between the intra-continental distributions of cholera epidemics and the dominant wind direction over land. Synoptic data can be an important indication for predicting the direction in which cholera is spreading, by daily monitoring and forecasting of wind direction and intensity.

Because cholera continues to be an important cause of morbidity and mortality in Africa, Asia, and Latin America, further investigations of aerial transfer may improve the crucial ability to predict its dissemination.

Interactions between climatologists, entomologists, and physicians are generally limited. They do not exchange information that can be readily used in vector management. By raising the awareness of the relationship between cholera outbreaks and synoptic conditions, disease spread could be halted before a full-blown epidemic develops. Today, the importance of such collaboration is increasing dramatically because human activity enhances the greenhouse effect, causing climatic changes (e.g., Gartell 2001; IPCC 2001). This trend has many global and local aspects, one of which is the impact on human health. Global climate change may affect human health via pathways of varying complexity, scale, directness, and timing. Similarly, the geographic impact may vary depending on the physical and environmental conditions and on the vulnerability of the local human population (Marsh and Gross 2001; McMichael 2003). Although most research deals with the influence of temperature increase on vector-borne diseases, it is important to study the impact of wind (direction and intensity) on spread of disease because wind is directly influenced by changes in atmospheric circulation. A good tool for monitoring and predicting cholera outbreaks will challenge a similar analysis of other pandemic diseases.

## Figures and Tables

**Table 1:** Deviation of mean monthly SLPs in March 1993 from the mean monthly values of March 1951–2000 at selected grid-points.

Selected grid-points	SD for March 1951–2000	March 1993 deviation	*t*	*p*-Value
15°00′N/75°00′E	0.94	1.67	1.773	0.041
20°00′N/75°00′E	1.12	1.99	1.769	0.041
20°00′N/87°30′E	1.11	1.85	1.671	0.050
22°30′N/75°00′E	1.15	2.21	1.916	0.030
22°30′N/80°00′E	1.32	2.23	1.682	0.049
25°00′N/75°00′E	1.26	2.38	1.897	0.032
25°00′N/87°30′E	1.45	2.73	1.887	0.033
27°30′N/80°00′E	1.63	2.76	1.692	0.049

Values are based on *t*-test results (*p* ≤ 0.05).
